# Acupuncture's Multisystem Neuroimmunomodulation: Central–Peripheral Interactions in Gastroenteric, Psychiatric, and Chronic Pain Disorders

**DOI:** 10.1111/cns.70625

**Published:** 2025-11-06

**Authors:** Lijuan Zhang, Yucai Luo, Ting Wei, Dan Wang, Zhaoxuan He, Lei Lan, Fang Zeng

**Affiliations:** ^1^ Acupuncture and Tuina School/The 3rd Teaching Hospital Chengdu University of Traditional Chinese Medicine Chengdu Sichuan China; ^2^ Acupuncture and Brain Science Research Center Chengdu University of Traditional Chinese Medicine Chengdu Sichuan China

**Keywords:** acupuncture, central‐peripheral interaction, humoral network, immune system, neural circuitry

## Abstract

**Background:**

Acupuncture, an ancient therapeutic modality rooted in traditional Chinese medicine, has evolved into a globally recognized intervention. Mounting evidence indicates that modulation of central–peripheral interactions constitutes a key pathophysiological mechanism underlying its efficacy. This review examines how acupuncture bridges neural, immune, and endocrine systems through these dynamic interactions to address complex modern diseases.

**Methods:**

We integrate cutting‐edge advances from neuroimaging and mechanistic studies to elucidate acupuncture's mechanisms targeting central‐peripheral crosstalk. Three paradigmatic conditions—functional gastrointestinal disorders, psychiatric disorders, and chronic pain—are employed to dissect its systemic effects.

**Results:**

Acupuncture consistently demonstrates therapeutic benefits across paradigms through multisystem modulation. Core mechanisms include: neural circuitry dynamics (brain‐gut/sensory axis regulation), neuro‐immune‐endocrine integration (cytokine‐HPA‐glial signaling), and humoral network modulation (hormone/metabolite‐mediated cross‐talk). These actions remodel complex pathophysiological networks, translating into improved clinical outcomes.

**Conclusions:**

Acupuncture emerges as an integrative therapy with a robust scientific foundation in systems biology. Future research leveraging omics and neuroimaging should focus on developing precision protocols targeting individual genetic, epigenetic, and connectomic profiles. This evolution holds significant promise for advancing integrative neurology and expanding the application of acupuncture against complex diseases.

## Graphical abstract

This work elucidates acupuncture's multisystem therapeutic mechanisms through central‐peripheral crosstalk. Using functional gastrointestinal disorders, psychiatric disorders, and chronic pain models, we demonstrate its efficacy in rebalancing gut‐brain signaling, neuroimmune‐endocrine integration, and humoral network modulation.

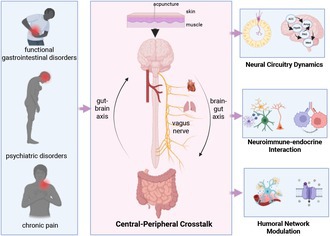

## 1 Introduction

Acupuncture is one of the world's oldest recognized medical treatments and has become widely accepted as a complementary and alternative therapy in modern healthcare. Its popularity is largely due to its low risk of adverse events when compared to many conventional treatments. This review explores the foundational principles and rich history of acupuncture, explains its underlying mechanisms of action, and evaluates its potential effectiveness in treating a range of diseases. In doing so, it highlights the growing body of research supporting acupuncture as a valuable adjunct to conventional medical practices.

### 1.1 Historical Evolution of Acupuncture

Acupuncture, a millennia‐old therapeutic practice originating in ancient China [1], encompasses both needling and moxibustion techniques. Its evolution reflects profound technological and theoretical advancements. Lithic Era (3000–2000 bc): Primitive *bian stones*—chiseled stone tools with sharpened edges—were employed for bloodletting and abscess drainage, as documented in the *Huangdi Neijing*. Bronze Age (1000–221 bce): Nine classical needles were bronze implements specifically designed for piercing, scraping, and incision [2]. Distinct from needling, moxibustion utilizes smoldering *Artemisia vulgaris* (mugwort) to deliver controlled thermal stimulation at acupoints. The selection of mugwort stems from its combustible properties. Low ignition temperature (160°C–200°C) enabling sustained smoldering without open flames. Other is the phytochemical profile. Volatile terpenoids (e.g., cineole, thujone) with demonstrated anti‐inflammatory and vasodilatory effects [3]. This bimodal approach—mechanical needling and thermal moxibustion—established acupuncture as a versatile therapeutic system, preserved through textual transmission (e.g., Zhenjiu Jiayi Jing) and sustained clinical practice.

### 1.2 Acupuncture Bridges Ancient Practice and Modern Neuroscience to Unravel the Biological Mechanisms of a Clinically Validated Therapy

**FIGURE 1:**
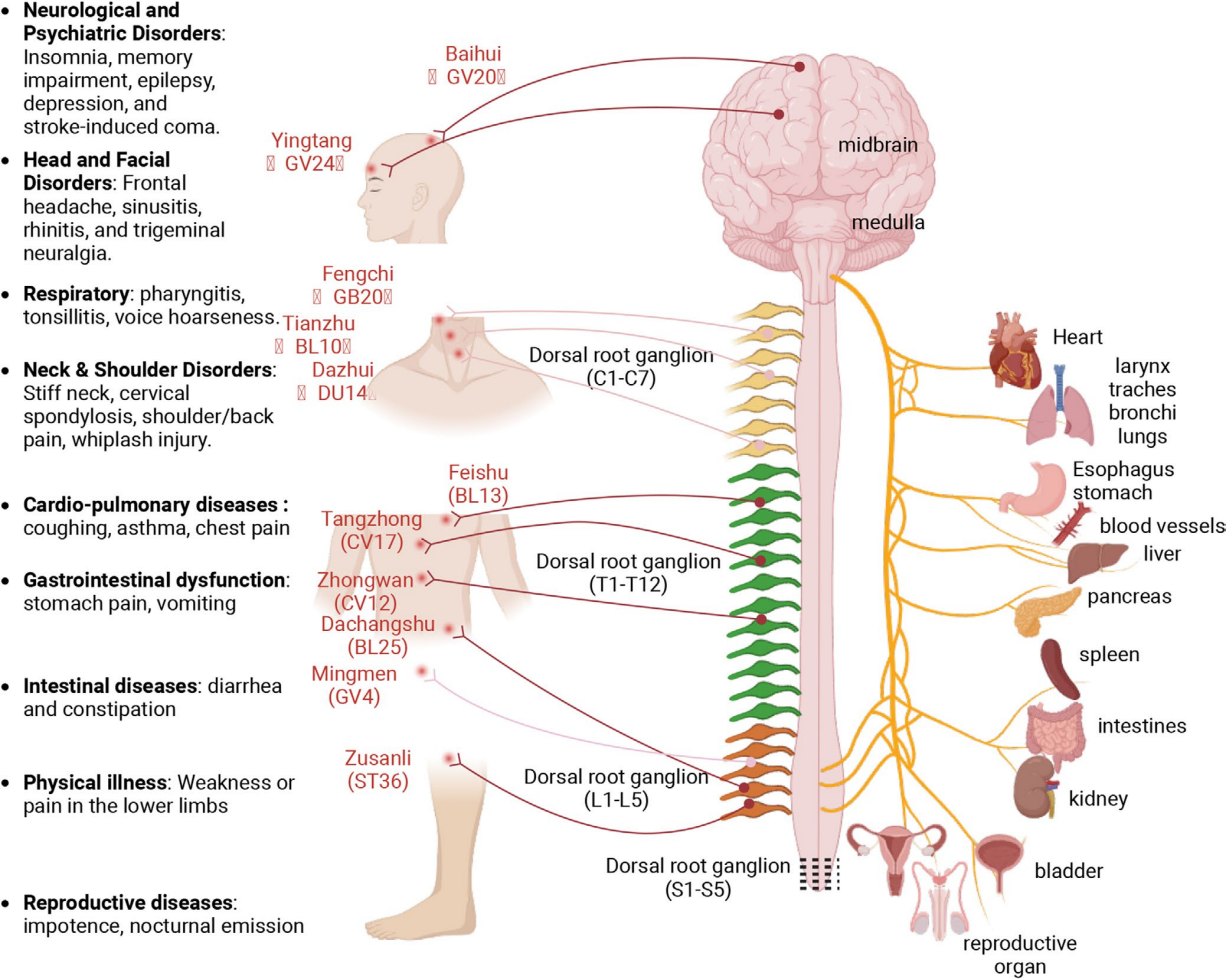
Neuro‐visceral circuit of acupoints: Disease targets, DRG segments, and visceral pathways. This schematic illustrates the tripartite relationship between: Clinical Applications (Left), Diseases treated by acupoints; Neural Segregation (Center), Corresponding dorsal root ganglion (DRG) segments acting as neural relays; Visceral Integration, DRG‐vagus nerve pathways mediating organ‐specific effects. Arrows indicate bidirectional communication between DRG levels (T1‐S5) and visceral organs through vagal afferent/efferent loops. The light‐colored arrows indicate the pathway from the dorsal acupoints through the vagus nerve to the corresponding stage in the DRG, while the dark‐colored arrows represent the pathway for the abdominal side acupoints. The figure was created with BioRender.com.

Acupuncture has been reported to be effective in the treatment of 307 diseases, of which more than 100 are highly effective [2]. Acupuncture stimulation is transmitted through peripheral nerve fibers to higher ganglia, which relay signals to the brain and modulate physiological responses, thus treating various diseases (Figure 1). From a traditional Chinese medicine perspective, this neural activation is believed to harmonize the flow of qi within meridians, facilitating balance and therapeutic effects through the interconnected network of energy pathways [3]. Acupuncture is often regarded as an alternative therapy or a pseudoscience in some countries because of the lack of sufficient modern medical trials to confirm its efficacy. The theory and practice of Chinese medicine are not based on Western medical research theories and are characterized as quackery from the point of view of some Western medical practitioners. Therefore, the biological mechanism of acupuncture for the treatment of diseases is still unclear and needs to be studied in depth.

## 2 Progress of Acupuncture Therapy in Diseases

**FIGURE 2:**
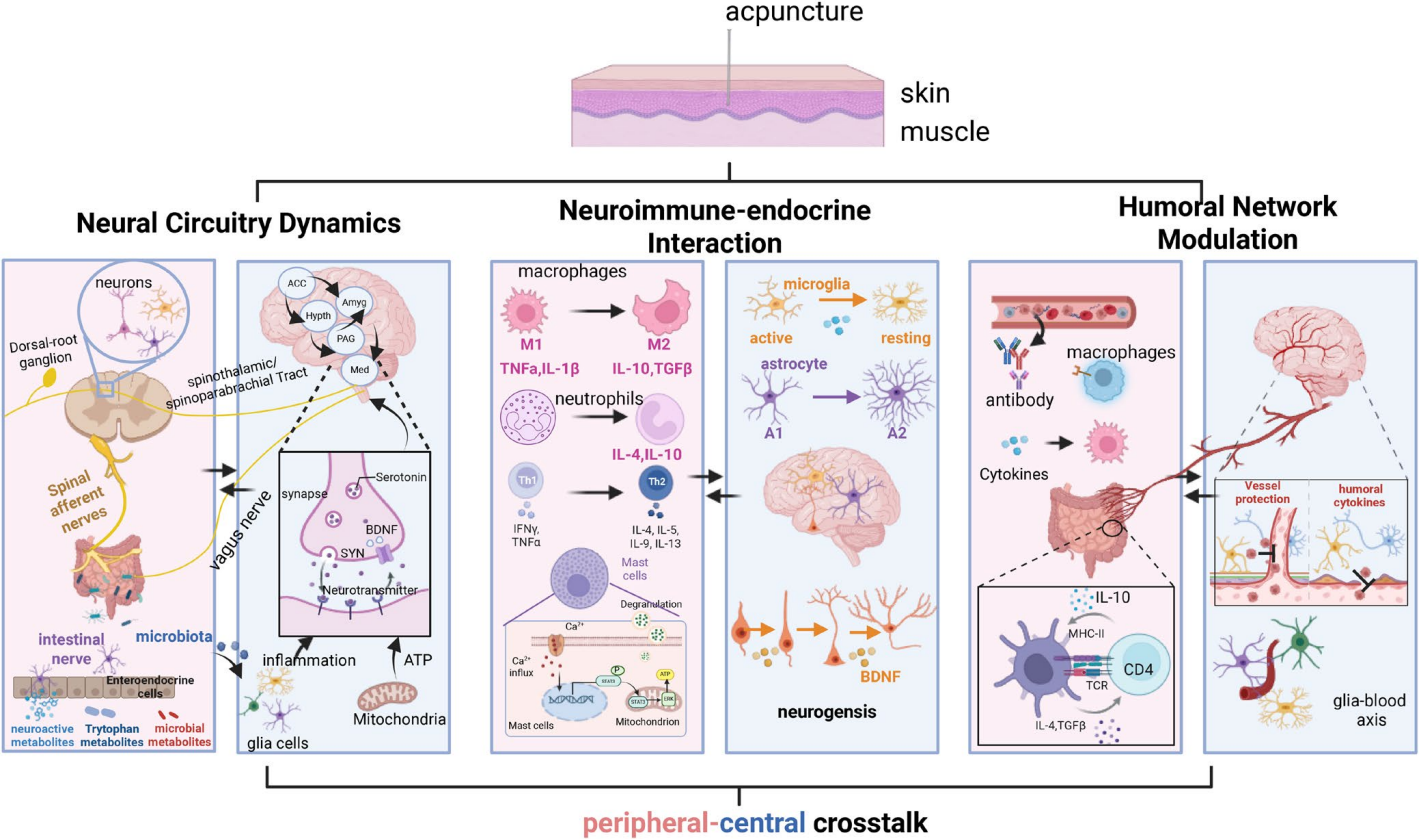
Peripheral‐central crosstalk: An integrative framework. Acupuncture exerts therapeutic effects through integrated peripheral‐central crosstalk spanning three mechanistic dimensions: Neural Circuitry Dynamics, Neuro‐immune‐endocrine Integration, Humoral Network Modulation. Focusing on peripheral‐central crosstalk, we reviewed the mechanisms of neural circuit dynamics, neurotransmitter modulation, inflammatory cell engagement, humoral release, sympathetic and parasympathetic systems, et al. The content of the pink background image is about the peripheral mechanism, while the content of the blue background image is about the central mechanism. Bidirectional arrows denote dynamic information flow between the periphery and CNS. BBB, blood–brain barrier; BDNF, brain‐derived neurotrophic factor; CNS, central neuron system; SYN, synaptophysin. The figure was created with BioRender.com.

Over centuries of evolution, acupuncture has become one of the most widely practiced traditional medical therapies worldwide. Celebrated for its safety, effectiveness, and minimal side effects, it is employed in the treatment of a broad spectrum of diseases, underscoring its significant clinical potential. However, the underlying mechanisms driving acupuncture's therapeutic effects are not yet fully understood, particularly in regard to its interactions with the central nervous system. As Figure 2 visually synthesizes, this research delineates central–peripheral crosstalk across three mechanistic dimensions: neural circuitry dynamics, neuro‐immune‐endocrine integration, and humoral network modulation. We empirically validated this framework using three disease paradigms—brain‐gut/sensory axis regulation, cytokine‐HPA‐glial signaling, and hormone/metabolite‐mediated cross‐talk—and further exemplified it with three representative pathologies: functional gastrointestinal disorders, psychiatric conditions, and chronic pain.

### 2.1 Modulating Gut‐Brain Neural Circuitry Dynamics in FGIDs

Functional gastrointestinal disorders (FGIDs) represent the most common diagnoses in gastroenterology, affecting over 40% of the global population [4]. These disorders significantly impact healthcare utilization and reduce patients' quality of life. The pathophysiology of FGIDs involves several factors, including alterations in gut microbiota, visceral hypersensitivity, changes in mucosal and immune function, and dysfunction of the central nervous system (CNS) [5]. Among FGIDs, functional dyspepsia (FD) is one of the most prevalent conditions, with a prevalence rate of 20%–40% [6]. According to the latest Rome IV criteria, FD is characterized by epigastric pain and burning sensations. These subtypes often overlap in clinical presentation [7]. The etiopathogenesis of FD remains incompletely understood in Western medicine, with current pharmacological interventions often providing suboptimal outcomes. This therapeutic gap has stimulated interest in alternative approaches that offer enhanced efficacy and reduced adverse effects. As a promising non‐pharmacological modality, acupuncture demonstrates clinical potential for FD management, with robust efficacy evidence and potential mechanisms summarized in Table 1. However, its mechanistic basis requires systematic elucidation. This study will investigate and synthesize the multilevel mechanisms through which acupuncture ameliorates FD pathology, focusing on brain‐gut axis modulation.

**TABLE 1:** Proposed clinical trials for acupuncture in FD (2015–2025).

Patient and diagnosis	Target mechanism	Treatment	Acupoints	Control group	Parameters	Primary endpoints	Mechanistic biomarkers	References
70 FD patients, aged 18–45 years old, Rome III criteria.	Brain function	MA	CV12 and ST36	Sham acupuncture	60–90 times per minute, after deqi response for 30 min, 20 sessions	Decreased the score of NDSI	fMRI. decreased the left BLA rsFC with bilateral insular, putamen and middle/posterior cingulate cortex, right pallidum and HIPP	[8]
60 FD patients, 18–40 years old, Rome III and IV criteria	Brain function	MA	ST36, BL21, and CV12	Sham acupuncture	20 sessions of acupuncture treatment over 4 weeks, 1–1.5 Hz, retained for 30 min	Lower baseline scores of the Symptom Index of Dyspepsia and longer durations of the condition	Critical predictive features	[9]
FD with EPS, 18–60 years, Rome III criteria	Mental status	MA	ST36, PC6, and RN12, or other points	Sham acupuncture	Different frequencies and times	Improvement of dyspeptic symptoms, quality of life, and mental status	NDI	[10]
132 FD PDS, 18–70 years, Rome III criteria	Mental status	EA plus on‐demand gastrocaine	ST34, ST36, ST40, ST42, CV12, PC6, BL20, and BL21	Sham acupuncture with Gastrocaine	20 sessions of EA over 10 weeks	Relief of FD symptom	Questionnaire	[11]
216 patients with FD, 18–50 years, Rome IV criteria	Vagal nerve and sympathetic nerve function	EA	RN12 and bilateral ST36	Sham acupuncture	60/100 Hz, 0.1–1.0 Ma, 4 weekly cycles of one treatment per day for 5 consecutive days	Mediated gastric motility	Reducing vagal nerve activation and inhibiting sympathetic nerve activity	[12]
200 Patients with refractory FD, 18–65 years, Rome III criteria	Mental status	EA	ST36, PC6, TR3, ST2, SP4, SP9	Sham acupuncture	20 sessions, duration of 4 weeks	Adequate relief of dyspeptic symptoms	LDQ, NDI, and adverse events	[13]

*Note:* Pishu (BL20); Weishu (BL21); Zhongwan (CV12); Neiguan (PC6); Erjian (RN12); Liangqiu (ST34); Zusanli (ST36); Feilong (ST40); Chongyang (ST42).

Abbreviations: BLA, basolateral amygdala; CMA, centromedial amygdala; DSSS, dyspeptic symptom sum score; EPS, epigastric pain syndrome; fMRI, functional magnetic resonance imaging; HIPP, hippocampus; LDQ, Leeds Dyspepsia Questionnaire; MRI, Magnetic Resonance Imaging; NDI, Chinese version of the Nepean Dyspepsia Index; NDSI, Nepean Dyspepsia Symptom Index; PDS, postprandial distress syndrome; rsFC, functional connectivity.

The gut‐brain axis is a bidirectional regulatory network that connects the gastrointestinal (GI) tract and the brain [14]. It comprises the CNS, autonomic nervous system, hypothalamic–pituitary–adrenal axis, and enteric nervous system [15]. This axis plays a critical role in regulating gastrointestinal hormones and, in turn, controls the sensory and motor functions of gastrointestinal smooth muscles [16]. Dysfunction of the gut‐brain axis is considered a major pathogenic mechanism of FD [17]. Studies have shown that imbalances in the gut‐brain axis, including dysregulation of brain‐gut peptide hormones and gut microbiota, can trigger FD [18]. Brain‐gut peptides, which are primarily secreted by endocrine cells in the gastrointestinal tract, include neuropeptides, gastrointestinal hormones, and other regulatory peptides [19]. Abnormal secretion or expression of these peptides can lead to gastrointestinal motility disorders and heightened visceral sensitivity, resulting in symptoms such as abdominal pain, bloating, and diarrhea [20].

The gut microbiota and the gut‐brain axis together form the microbiota‐gut‐brain axis, which plays a crucial role in regulating the gut‐brain axis and maintaining its homeostasis in the body [21]. Studies have shown that the gut microbiota of patients with FD undergoes significant alterations compared to healthy individuals [22]. Acupuncture may exert its therapeutic effects by influencing the descending pathways of the gut‐brain axis, thereby regulating the gut microbiota, improving the secretion of brain‐gut peptides, modulating local inflammatory responses [23], alleviating gastrointestinal dysfunction [24], and repairing gastric mucosal damage in FD patients [25]. At the same time, impaired gastrointestinal function can lead to significant changes in the hypothalamic–pituitary–adrenal (HPA) axis. The HPA axis plays a regulatory role in both brain and gastrointestinal functions through the secretion of corticotropin‐releasing factor [26]. Research has demonstrated that in FD rat models, increased gastric hypersensitivity and impaired gastric motility are associated with elevated central hormone expression and dysregulation of the HPA axis, a mechanism mediated by hormone receptors [27]. In rat models of irritable bowel syndrome, EA treatment has been shown to reduce hypothalamic hormone expression and repair the intestinal mucosal barrier [28].

More importantly, due to the development of functional Magnetic Resonance Imaging, brain functional imaging technology provides a visual and objective research approach to study the brain response mechanism of acupuncture treatment of FD. Acupuncture along meridians can induce the response of specific brain regions in the central nervous system, which is one of the important scientific evidences that acupuncture regulates brain‐gut interaction in the treatment of FD. Previous research by our group identified that acupuncture stimulation can inhibit glucose metabolism in the insula, thalamus, brainstem, and hypothalamus of FD patients [29, 30]. Over the past decade, numerous neuroimaging studies have shown that FD patients show significant functional and structural changes in multiple brain regions [31, 32]. Our laboratory has long been committed to the study of the functional mechanism of acupuncture in the treatment of FD. Our study aimed to investigate the influence of acupuncture stimulation on brain activities in FD patients. The results showed that the anterior cingulate cortex (ACC), prefrontal cortex, and caudate tail were involved in gastric sensory processing. The inactivation of the primary somatosensory area and cerebellum was related to acupuncture stimulation. Activation of the visual‐related cortex is a response to pain or acupoint action [29]. Similarly, recent studies showed that the amplitude of low‐frequency fluctuation induced higher spontaneous brain activity in the primary motor/sensory areas and homeostatic‐afferent network regions [31]. In our previous study, we investigated the effects of acupuncture on brain glucose metabolism and explored the possible correlation between brain response and clinical efficacy. The results showed that acupuncture had a more significant regulatory effect on the homeostatic afferent network, including the insula, ACC, and hypothalamus [29]. In the subsequent study, we deeply explored the similarities and differences of brain region responses in treating FD with acupuncture at different points; the study indicated that the clinical effect of different acupoints is similar, but the brain response is different [33]. However, the mechanism by which different acupuncture points respond differently to the brains of FD patients is still unclear. Neuroimaging evidence reveals acupoint‐specific neural circuit engagement. Stimulation of Zhongwan (CV12) predominantly recruits the postcentral gyrus‐reward network circuit. Stimulation of ST36 primarily activates the postcentral gyrus‐default mode network (DMN) pathway [34]. Notably, these circuits exhibit direct pathophysiological relevance to FD, where structural and functional abnormalities in the precuneus—a core DMN hub—are consistently observed in FD patients [35, 36]. This convergence suggests that acupoint selection may specifically target disease‐relevant network pathologies. More importantly, a number of studies have reported that functional connectivity between the sensorimotor network and DMN after acupuncture treatment in FD patients was different from healthy controls [37]. Our imaging data also showed that the regulation of the right medial prefrontal cortex (mPFC) function and its connectivity with DMN is one of the important mechanisms in the treatment of FD by acupuncture [38].

Acupuncture has been shown to stimulate the secretion of gastrointestinal neuropeptides associated with gastrointestinal motility, effectively restoring normal gastrointestinal function [39]. This suggests that acupuncture can alleviate clinical symptoms of FD by modulating brain‐gut peptides. For example, electroacupuncture (EA) treatment has been shown to significantly improve gastrointestinal motility in FD rat models, along with increased expression of ghrelin in GI tissues, indicating its therapeutic effect through hormone regulation [40]. Furthermore, research has demonstrated that EA intervention in FD rats reduces the expression levels of intestinal peptide genes in GI tissues. This downregulation effectively suppresses abnormal GI motility and decreases the incidence of inflammatory GI diseases [41]. Additionally, serotonin (5‐HT) plays a crucial role in promoting GI motility, regulating colonic movement, and modulating visceral hypersensitivity. It is also critical in maintaining gut and brain barrier integrity [42]. Data indicate that EA significantly increases 5‐HT expression in FD rat models, promoting the restoration of normal GI motility and markedly reducing visceral sensitivity [43]. These findings highlight the potential of acupuncture, particularly EA, as a therapeutic approach to modulating the gut‐brain axis and improving FD symptoms.

### 2.2 Regulating Neuro‐Immune‐Endocrine Integration in Psychiatric Disorders

Depression, as one of the most severe mental illnesses, is a leading cause of disability worldwide [44]. According to the World Health Organization, it is projected to become the primary contributor to the global disease burden by 2030 [45]. Clinical symptoms of depression include anhedonia, insomnia, and fatigue, which often result in poor responses to pharmacological treatments [46]. Acupuncture represents a cost‐effective therapeutic modality with a favorable safety profile, demonstrating significant clinical utility across the depression spectrum—from mild to severe cases, including treatment‐resistant variants and comorbid conditions. As summarized in Table 2, robust efficacy evidence and emerging mechanistic insights support its role in depression management. Nevertheless, the precise neurobiological mechanisms underlying the antidepressant effects of acupuncture require further elucidation. This study will decipher the multi‐level antidepressant mechanisms of acupuncture.

**TABLE 2:** Proposed clinical trials for acupuncture in depression (2015–2025).

Patient and diagnosis	Target mechanism	Treatment	Acupoints	Control group	Parameters	Primary endpoints	Mechanistic biomarkers	References
304 participants, moderately severe depression, 18–50 years	Mental status	MA	Auricular acupuncture, auricular pavilion	Nonspecific AA	3 times a day, 6 weeks	Decrease the score of PHQ‐9, and depression remission	PHQ‐9	[10]
270 patients with insomnia in patients with depression, 18–70 years	Mental status	EA	GV20, GV24, GV29, EX‐HN22, HT7, PC6, and SP6	Sham acupuncture	30‐min treatment 3 times per week for 8 weeks	Reduction of severity of insomnia, depressive mood, and anxiety symptoms	Mental State and Sleep Quality	[47]
12 healthy participants and 30 patients with medication‐resistant depression, ICD‐10	Vagal function	Press needle acupuncture	PC4, LI10, SP9, SP6	Sham‐ press needle	72 h	The alterations in vagal function, blood pressure, and Beck's Depression Inventory scores	improve the vagal function	[48]
60 patients with moderate depression, 18–70 years, DSM‐IV	Metabolic alterations	EA	GV20, GV29, GV24, and HT7, PC6, ST36, and SP6	Sham acupuncture	10 Hz, 30 min per session, 3 times per week, for 8 weeks	Scores of HAMD decreased	tryptophan metabolism, glutamate metabolism, and fatty acid biosynthesis	[49]
121 Women diagnosed with PPD, 20–49 years, DSM‐IV	Anti‐inflammation	MA	GV20, GV29, CV12, CV6, CV4, PC6, HT7, LI4, SP6, and LR3	Sham acupuncture	30 min for 8 weeks, twice weekly, 16 sessions	Score of HDRS‐17 decreased	IL‐5, TNF‐α and TGF‐β1; IL‐6, IL‐10 and IFN‐γ	[50]
60 patients with mild perimenopausal depression	Mental Status	MA	GV 26, LU 11, SP 1, PC 7, BL 62, ST 6, CV 24, PC 8, GV 23	Sham acupuncture	Once every other day, 3 times a week for 12 weeks	HAMD, SDS and Kupperman scores were all reduced	HAMD, SDS and Kupperman	[51]
163 depression, 18–70 years, CCMD‐3	Mental Status	MA	GV20, GV29, LI 4 and LR 3, BL 17, BL 19), BL 15, BL 18	Sham acupuncture	2 times a week, 12 weeks	Scores on SF‐36 were decreased	SF‐36	[52]
161 moderate to severe depressed patients, 18–60 years, DSM‐V	Mental Status	MA, EA, SSRIs	GV20, GV29, GV16, GB20, GV14, PC6, SP6	Sham acupuncture	2–15 Hz, at different weeks	HAMD‐17 score decreased	HAMD‐17	[53]
120 participants with MDD, 18–60 years, DSM‐V	Brain functions	Intradermal acupuncture	HT7, PC6, SP6 and LR3	Sham acupuncture	10 sessions over 6 weeks	HAMD‐17 scores decreased	fMRI, normalization of functional connectivity in striatum and cerebellum	[54]
70 eligible patients, 18–65 years, DSM‐V	Brain functions	MA	Soothing liver‐qi stagnation, Tonify and invigorate the heart and the spleen Yang	Sham acupuncture	twice per week for 3 consecutive weeks	HAMD‐17 and PHQ‐9 score was decreased	fMRI, normalization of functional connectivity in the dorsolateral prefrontal cortex	[55]
56 female MDD, 30–60 years, ICD‐10	Brain functions	MA plus fluoxetine	RN12, RN10, RN6, RN4, KL17, ST24	Sham acupuncture plus fluoxetine	Once a day for the first 3 days and once every 3 days for the remainder 8 weeks	MADRS and the SDS score	fMRI, normalization of functional connectivity in the DMN, AN, SN, and CCN is normal	[56]
56 pregnant or breastfeeding women, 30–60 years, ICD‐10	Brain functions	MA plus fluoxetine	RN12, RN10, RN6, RN4, KL17, ST24	Sham acupuncture plus fluoxetine	Once a day for the first 3 days and once every 3 days for the remainder 8 weeks	Clinical improvement as indicated by MADRS and SDS scores	fMRI, increase of functional connectivity in the left amygdala and sgACC/pgACC	[57]

*Note:* Baihui (GV20), Shenting (GV24), Yintang (GV29), Anmian (EX‐HN22), Shenmen (HT7), Neiguan (PC6), and SanYinjiao (SP6), ZuSanLi (ST36), Zhongwan (CV12), Qihai (CV6), Guanyuan (CV4), Neiguan (PC6), Hegu (LI4), Taichong (LR3), Shuigou (GV 26), Shaoshang (LU 11), Yinbai (SP 1), Daling (PC 7), Shenmai (BL 62), Jiache (ST 6), Laogong (PC 8), Shangxing (GV 23), Geshu (BL 17), Danshu (BL 19), Xinshu (BL 15), Ganshu (BL 18), Fengfu (GV16), Fengchi (GB20), Dazhui (GV14), Zhongwan (RN12), Xiawan (RN10), Qihai (RN6), Guanyuan (RN4), Shangqu (KL17), Huaroumen (ST24).

Abbreviations: AA, Auricular acupuncture; AN, affective network; CCMD‐3, China Society of Psychiatrics, the third edition; CCN, cognitive control network; DMN, default mode network; DSM‐IV, Diagnostic and Statistical Manual of Mental Disorders, Fourth Edition; FC, functional connectivity; fNIRS, Functional Near‐Infrared Spectroscopy; HAMD, Hamilton Depression scale; HDRS‐17, Hamilton Depression Rating Scale; HPA, hypothalamic–pituitary–adrenal; ICD‐10, International Classification of Disease, 10th edition; MADRS, Montgomery‐Åsberg Depression Rating Scale; MDD, major depressive disorder; pgACC, preguenual anterior cingulate cortex; PHQ‐9, Patient Health Questionnaire‐9; PPD, postpartum depression; PSQI, Pittsburgh Sleep Quality Index; SDS, self‐rating depression scale; SDS, Self‐Rating Depression Scale; sgACC, subgenual anterior cingulate cortex; SN, salience network; SSRIs, selective serotonin reuptake inhibitors; TNF‐α, tumor necrosis factor‐α.

Although the cause of depression is unknown, the brain plays an important role in the pathogenesis of depression. Changes in neural circuits, or the brain's chemical balance, are thought to be physiological abnormalities that directly lead to depressive symptoms [58]. A large number of neuroimaging studies have found abnormal structure and function in specific brain regions or connections that are involved in cognitive and emotional processes, including the hippocampus [59]. Our study showed that the reduction in hippocampal volume of depression model mice, was related to the duration of depression, and herb medicine can effectively mitigate this decrease [60]. Other brain areas also could affect the symptoms of depressive disorder, such as mPFC, ACC, amygdala, and accumbens nucleus [61, 62]. Acupuncture may act in a variety of ways, such as biochemical synthesis, neuronal structures, and stimulating neuronal circuits.

The monoamine neurotransmitter 5‐HT plays an important role in synaptic plasticity [63]. Studies suggest that a decrease in monoamine neurotransmitter levels in the brain may trigger depression [64]. Research on acupuncture treatment in depressed rat models has shown that acupuncture increases the levels of monoamine neurotransmitters in the hippocampal tissue, alleviates neuronal structural damage, and improves depressive behaviors in rats [34]. A study used depressive model rats to explore the possible mechanism of EA on the hippocampal CA1 region neuronal synaptic plasticity; the result showed that EA could ameliorate depressive‐like behaviors by restoring synaptic plasticity, mediated by regulating 5‐HT receptor levels [65]. Analysis of 25 brain regions involved in the regulation of 5‐HT1A receptor activation, including the cortex, hippocampus, thalamus, and hypothalamus, revealed that the activation of 5‐HT1A receptors was significantly reduced in depression model rats. However, acupuncture was found to enhance the expression of 5‐HT1A receptors in the brain, alleviating depressive symptoms [66]. This indicates that acupuncture has a regulatory effect on monoamine neurotransmitter levels and may exert its antidepressant effects by modulating 5‐HT receptors.

A ubiquitous neurotrophic factor, brain‐derived neurotrophic factor (BDNF), is essential for the development of the nervous system [67]. Our study also showed that increasing the content of BDNF could increase the stress resistance of depressed model mice [68]. Using a rat model of depression, the study demonstrated that EA at the Baihui (GV20) and Yintang (GV29) acupoints could significantly increase the concentrations of BDNF mRNA in the hippocampus [69]. In addition, another study found that EA increased BDNF levels by modulating multiple targets in the cyclic adenosine monophosphate response element binding protein signaling pathway, thereby promoting nerve regeneration and playing an antidepressant role [70]. In summary, acupuncture can prevent the reduction of BDNF levels, thereby improving depressive symptoms.

The “inflammatory response system model of depression” suggests that depression is associated with the activation of inflammatory responses [71]. Pro‐inflammatory cytokines act on the central nervous system, leading to behavioral, neuroendocrine, and neurobiochemical changes associated with depression [72]. The NLRP3 inflammasome is expressed in key brain regions related to depression and is considered a potential biomarker of the disorder [73]. Upon stimulation, the NLRP3 inflammasome becomes activated, promoting the release of mature inflammatory cytokines, initiating inflammatory responses, and contributing to the development of depression. Acupuncture has been shown to inhibit the activation of the NLRP3 inflammasome, reduce the release of inflammatory cytokines, alleviate inflammatory responses, and improve depressive symptoms [74]. For instance, after six weeks of acupuncture at GV20 and ST36 points in patients with depression, symptoms improved along with a reduction in IL‐1β and IL‐6 levels [75]. Microglia, the primary immune cells in the central nervous system, play a crucial role in regulating the immune system in patients with depression [76]. Chronic activation of microglia can lead to immunotoxicity, characterized by the excessive production of neurotoxic substances, including inflammatory cytokines, nitric oxide, and peroxides, which ultimately exacerbate neuronal damage [77]. Positron emission tomography scans have shown that during cognitive behavioral therapy in patients with major depressive disorder, microglial activity in cortical and subcortical regions significantly decreased, along with changes in the expression of inflammation‐related genes in treatment groups [78]. Mechanistic studies demonstrate that acupuncture reverses microglial hyperactivation in chronic restraint stress (CRS) models by suppressing TLR4/MyD88 signaling and inhibiting NLRP3 inflammasome assembly. This process reduces serum levels of inflammatory cytokines such as IL‐1β, alleviates hippocampal apoptosis, and improves depressive symptoms [79]. The TLR4‐NLRP3 axis thus constitutes a critical neuroimmune switch targeted by acupuncture for mood disorder intervention. These findings indicate that acupuncture may improve depression by modulating the NLRP3 inflammasome and inhibiting its activation.

Patients with depression often exhibit hyperactivity of the HPA axis, characterized by elevated levels of HPA axis‐related hormones such as corticotropin‐releasing hormone, adrenocorticotropic hormone, and cortisol [80]. Acupuncture has been shown to enhance the activity of glucocorticoid receptors (GR), aiding in neuronal repair. However, whether this contributes directly to the normalization of HPA axis function requires further investigation [42]. In studies involving depressed rat models, acupuncture significantly reduced levels of hormones, along with improvements in behavioral indicators [81]. A review also highlighted that transcutaneous auricular vagus nerve stimulation (taVNS) possesses anti‐inflammatory properties by activating specific brain regions or circuits of the hypothalamic axis in patients with depression. It emphasized taVNS's ability to regulate the activity and connectivity of various neural networks effectively [82]. These findings suggest that acupuncture may improve depression by addressing feedback dysregulation within the HPA axis.

### 2.3 Restoring Humoral Network Homeostasis in Chronic Pain via Peripheral‐Central Desensitization

Among the more than 40 clinical indications for acupuncture outlined by the World Health Organization, pain is one of the most extensively studied and widely researched conditions. Acupuncture is applicable to various types of pain, especially chronic pain. Chronic pain is defined as pain that persists for more than three months, beyond the typical healing period, and is often associated with pathophysiological changes in the central nervous system. It can also lead to complex interactions between emotional, psychological, and social factors, significantly affecting both physical and mental health [83]. Patients with chronic pain may experience emotional disturbances, sleep disorders, and impaired social interactions [84]. One study treating chronic pain patients with EA at GV20 and GV29 points found that the treatment not only reduced pain perception but also alleviated depressive symptoms, thus relieving chronic pain [85]. Based on its etiology, chronic pain can be categorized into nociceptive pain (resulting from tissue injury or inflammation) and neuropathic pain (caused by primary damage or dysfunction in the nervous system) [84]. The former is triggered by the activation of nociceptors, while the latter stems from damage or dysfunction within the neural structures themselves. Currently, the clinical management of pain primarily relies on nonsteroidal anti‐inflammatory drugs and opioid analgesics [86]. Acupuncture exerts analgesic effects primarily through bidirectional peripheral‐central modulation under pathological conditions. Stimulation of acupoints triggers peripherally originated signaling and centrally initiated regulation, as summarized in Table 3; these mechanisms translate to clinically significant pain relief in chronic conditions. Building on this evidence, our study will specifically decode the humoral network homeostasis underlying the efficacy of acupuncture in cervical radicular pain and peripheral neuropathic pain models.

**TABLE 3:** Proposed clinical trials for acupuncture in chronic pain (2015–2025).

Pain type	Patient and diagnosis	Target mechanism	Treatment	Acupoints	Control group	Parameters	Primary endpoints	Mechanistic biomarkers	References
Fibromyalgia	65 participants with fibromyalgia, 26–64 years American College of Rheumatology criteria	Brain function	MA	DU20, LI11, LI4, GB34, ST36, SP6	Sham acupuncture	18 treatments over 12 weeks	Lower pressure pain thresholds	Meaning of Illness Questionnaire	[87]
Chronic musculoskeletal pain	360 cancer survivors with chronic musculoskeletal pain, about 60 years old	Peripheral function	EA or auricular acupuncture	Acupuncture points in tables	Sham acupuncture	2 Hz, 10 treatments over 10 weeks	Improved pain severity, pain‐related functional interference, and quality of life	Brief Pain Inventory	[88]
Chronic low back pain	59 participants with chronic low back pain, 21–65 years, disc compression or spinal stenosis	Peripheral function	EA	GV20, GV3, SI3, BL40, KI7, KI3, and so on	Sham acupuncture	45 min per session, 2 sessions per week for 6 weeks.	Reduction in RMDQ	RMDQ	[89]
Chronic neuropathic pain	60 chronic neuropathic pain, 18–75 years, North American Spine Society diagnostic criteria	Brain function	MA	GB30, GB3, GB33, GB34, GB39, BL36, BL40, BL54, BL57, and BL60	Sham acupuncture	10 sessions of treatment over 4 weeks	The VAS for leg pain and ODI score decreased after treatment	fMRI, neural activity of the right SPL and right postcentral gyrus	[90]
Neck/shoulder stiffness	400 patients with neck/shoulder stiffness, 18–60 years	Peripheral function	MA	BL10, GB21, SI14, BL42	Superficial skin piercing	24 h	Neck/shoulder stiffness	/	[91]
Chronic neck pain	99 patients with chronic neck pain, 18–65 years	Raphe nucleus‐related brain circuits	MA	SJ15, SI14, SI15, BL11, LI16	Sham acupuncture	3 times weekly for 4 weeks with a total of 12 sessions.	Improvements in pain‐related emotional impairment and quality of life	fMRI, changes in the DR‐related circuit, and MR‐related circuit	[92]
Chronic shoulder pain	24 patients with chronic shoulder pain, 45–65 years	Brain functional network	MA	ST38	Contralateral acupoint ST 38 or ipsilateral acupoint ST 38	20 min	Improving shoulder function and alleviating pain	fMRI, increased degree centrality in the anterior/paracingulate cortex	[93]
Chronic low back pain	79 patients diagnosed with chronic low back pain	mPFC‐insula/basal ganglia rsFC	MA	GV3, BL23, BL40, KI3	Streitberger needle	Twice a week for 2 weeks and then once a week for 2 weeks	Reduced PROMIS sub‐scores in pain intensity, physical disability and pain interference	fMRI, the FCs between the mPFC and the insula, putamen, caudate, and angular gyrus	[94]
Chronic spinal pain	50 volunteers with chronic spinal pain, 30–64 years	Peripheral function	Auriculotherapy	TF4, CO10, AH6a, CO9, CO12, AT1, AH13, AH11, AH9	Sham group	100 mW, 15 days	Improvement in pain intensity and pain threshold	Numeric Pain Rating Scale. A digital dynamometer model.	[95]
Chronic low back pain	102 chronic low back pain, 18–60 years, Quebec Task Force Classification System categories I‐II	Brain function	EA	GV3, BL23, BL40, KI3	Streitberger needles	2 Hz, 20 min, 6 treatments over 4 weeks	Improvements in pain	fMRI, reduced S1‐back gray matter volume and increased S1‐back adjacent white matter	[96]
CP/CPPS	100 patients with CP/CPPS, (category III B), 22–49 years	Sacral nerve	MA	BL33, BL34, BL54, CV1, CV4, SP6, SP9	Sham group performed 1 cm left of each selected acupoint	Every week for a period 6 weeks	Decrease in total NIH‐CPSI score	Stimulation of segments of sacral nerve	[97]

*Note:* Baihui (GV20), Yaoyangguan (GV3), Houxi (SI3), Yanglingquan (BL40/SP9), Fuliu (KI7), Taixi (KI3), Zhongliao (BL33); Xialiao (BL34); Zhibian (BL54); Huiyin (CV1); Guanyuan (CV4); Sanyinjiao (SP6); Tianliao (SJ15); Jianwaishu (SI14); Jianzhongshu (SI15); Dazhu (BL11); Jugu (LI16); Yaoyangguan (GV3); bilateral Shenshu (BL23); Weizhong (BL40); Tiaokou (ST38); Baihui(DU20); Quchi (LI11); Hegu (LI4); Yangmingquan(GB34); Zusanli (ST36); Sanyinjiao (SP6); Shenmen (TF4); Huantiao (GB); Fengshi (GB31); Xiyangguang (GB33); Xuanzhong (GB39); Chengshang (BL36); Weizhong (BL40); Xubian (BL54); Kunlun (BL60).

Abbreviations: ACC, anterior cingulate cortex; BDI, Beck Depression Inventory; CP/CPPS, chronic prostatitis/chronic pelvic pain syndrome; DR, dorsal; mPFC, medial prefrontal cortex; MPQ, McGill Pain Questionnaire; MR, median; NDI, Neck Disability Index; NIH‐CPSI, Health Chronic Prostatitis Symptom Index; NPQ, Northwick Park Neck Pain Questionnaire; ODI, oswestry disability index; RMDQ, Roland Morris Disability Questionnaire; rsFC, resting‐state functional connectivity; rsFC, resting‐state functional connectivity; SAS, Self‐rating Anxiety Scale; SDS, Self‐rating Depression Scale; SF‐12, 12‐item Short Form Quality Life Scale; SPL, superior parietal lobule; VAS, visual analog scale.

A functional magnetic resonance imaging study has found that cervical spine pain can lead to abnormal activation in multiple brain regions and alterations in brain network connectivity. This not only activates pain centers but also engages cognitive and emotional brain areas [98]. When acupuncture is applied to patients, similar responses occur across various brain lobes, suggesting a potential connection between functional regions of the brain, thereby exerting a central regulatory effect on the disease [99]. Research has shown that the sensory pathways activated during acupuncture stimulation are similar to those involved in pain perception. Peripheral nerves in the stimulated area transmit sensory signals to the dorsal horn of the spinal cord, where the signals continue along the anterolateral columns, which are responsible for transmitting nociceptive (pain and temperature) information, and then ascend to the cerebral cortex [100, 101]. The use of functional magnetic resonance imaging can help validate this mechanism. One study observed changes in the brains of patients after acupuncture. The results revealed significant responses in the sensory areas of the cortical regions. Compared to the control group, the homogeneity and functional connectivity in the sensory‐motor regions of the brain were reduced, providing clinical evidence of acupuncture's central regulatory mechanism in pain relief [102]. Further, the studies on EA treatment in a brachial plexus pain rat model found that acupuncture induced a reduction in dynamic causal modeling of the limbic–cortical feedback system. The study showed that during pain induction or acupuncture treatment, the sensory cortex, amygdala, and hypothalamus—all parts of the limbic system—were activated. These findings suggest that the organization of the cortical–limbic network may be linked to acupuncture‐induced analgesia. Acupuncture appears to influence the connectivity between the hypothalamus, amygdala, and the cortical sensory areas, which may represent a cortical mechanism for alleviating pain. Thus, it can be concluded that acupuncture exerts its effects on brain regions by modulating the central nervous system functions related to pain [103]. It regulates the connectivity between different functional areas of the brain and alleviates pain by stimulating conduction pathways similar to those activated by pain. This suggests that acupuncture plays a role in central pain modulation.

Acupuncture also acts on the peripheral nervous system. Numerous studies have shown that acupuncture can modulate the activity of neurons in central structures such as the dorsal root ganglion, spinal dorsal horn, nucleus of the solitary tract, thalamus, and cerebral cortex, as well as influence neurotransmitters in both the central and peripheral nervous systems, including 5‐HT, central opioid peptides, norepinephrine, and acetylcholine, thereby alleviating pain symptoms [104]. Research has indicated that acupuncture can relieve pain caused by nerve injury and improve sensory and motor functions [105]. TRPV1, a subtype of the transient receptor potential ion channel, is expressed in nociceptive neurons in the dorsal root ganglion and spinal dorsal horn. When these neurons are exposed to harmful stimuli, they transmit pain signals to the spinal dorsal horn and the trigeminal spinal tract, generating pain perception. In an inflammatory pain rat model, acupuncture at bilateral ST36 significantly reduced TRPV1 expression in the spinal cord and alleviated pain symptoms. This suggests that acupuncture can alleviate pain by downregulating the expression and sensitivity of TRPV1 [106]. Mast cells serve as primary mechanosensory transducers in acupuncture‐mediated immunomodulation. In acute adjuvant arthritis models, acupuncture stimulation directly activates perivascular mast cells, initiating canonical NF‐κB signaling through IκBα phosphorylation. Crucially, these cells convert mechanical stimuli into biochemical messengers, converting the physical stimulus from acupuncture into biochemical signals such as ATP, histamine, and encephalin, which influence humoral network homeostasis [107]. In CFA‐treated rats, daily EA stimulation at the ST36 acupoint reduced the levels of substance P, neurokinin‐1 receptor, and interleukin‐1β in the dorsal root ganglion [108]. These molecules are associated with pain signaling and inflammation, indicating that EA helps alleviate peripheral pain and inflammation. Acupuncture has been shown to relieve pain caused by peripheral nerve injury by increasing the pain threshold at the site of peripheral nerve lesions, reducing hyperalgesia, activating immune cells, and modulating ion channel expression. These effects help mitigate pathological nerve damage, such as demyelination, neuronal degeneration, and axonal degeneration, which can lead to neuropathic pain.

### 2.4 Acupuncture as a Systems Neuromodulator–Bridging Mechanistic Insights Toward Precision Integrative Medicine

**FIGURE 3:**
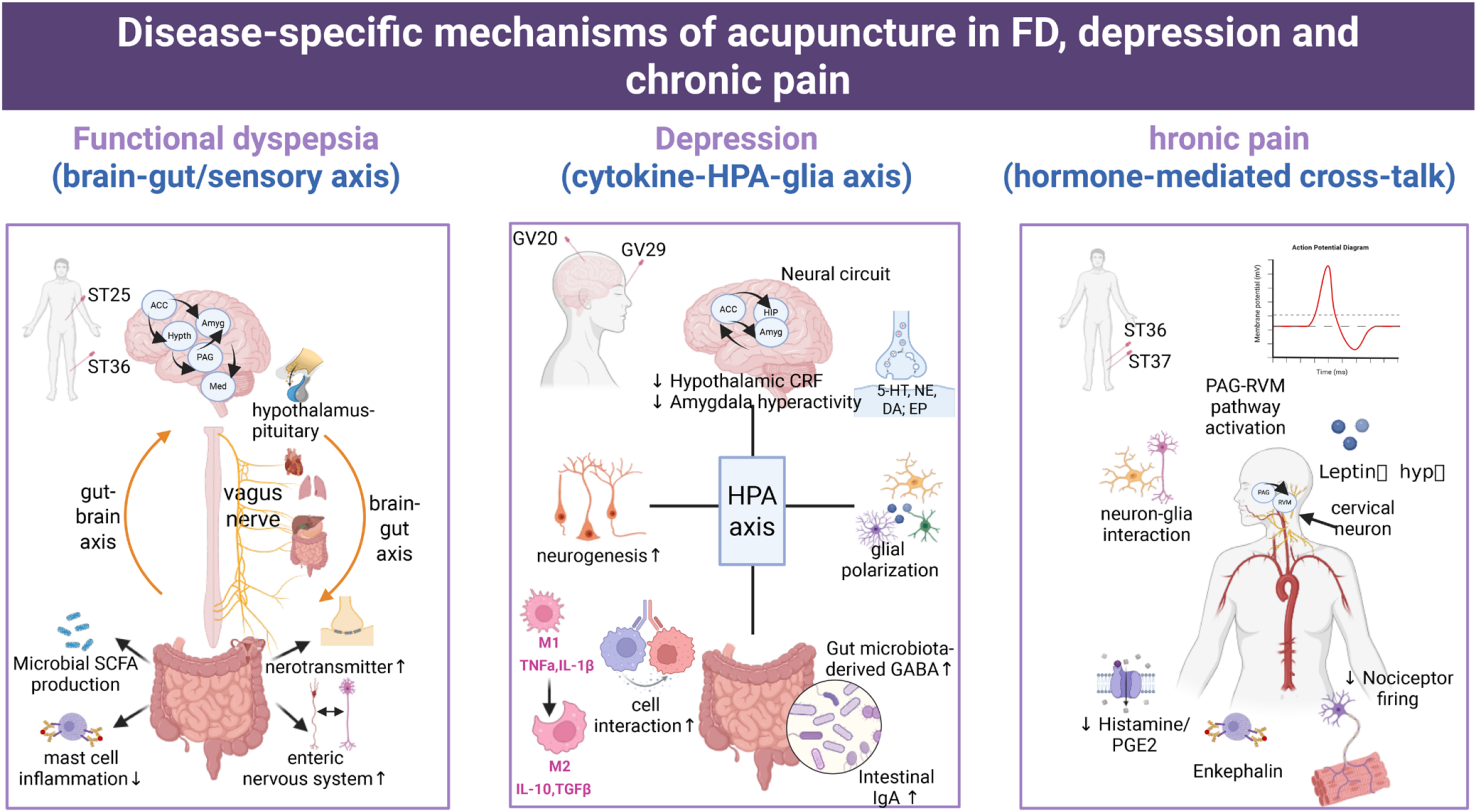
Disease‐specific mechanisms of acupuncture in FGIDs, depression, and chronic pain. Acupuncture exerts therapeutic effects through distinct peripheral‐central pathways: (left) FD treatment targets vagus‐mediated gut‐brain crosstalk via ST25, ST36 stimulation, modulating neural circuitry. Enteric glial‐driven motility normalization, microbial SCFA‐immune homeostasis, and vagal control. (middle) Depression management at GV20/GV29 resets the HPA‐immune‐microbiota axis, involving: The reduction of hypothalamic CRF, amygdala hyperactivity, microglial polarization and the increase of hippocampal neurogenesis, Gut GABA, and intestinal IgA. (right) Chronic pain therapy at ST36, ST37 reverses peripheral‐central sensitization through PAG‐RVM descending inhibition, ACC‐S1 synaptic remodeling. The inhibition of humoral release, histamine/PGE2 release, decreases the nociceptor firing. 5‐HT, 5 hydroxytryptamine; ACC, anterior cingulate cortex; Amyg, amygdala; CRF, corticotropin‐releasing factor; DA, dopamine; EP, endorphin; FD, functional dyspepsia; GABA, gamma‐aminobutyric acid; GV20, baihui; GV29, yingtang; HIP, hippocampus; HPA, hypothalamic–pituitary–adrenal axis; Hypth, hypothalamus; Med, medbrain; NE, norepinephrine; PAG, periaqueductal gray; PGE2, prostaglandin E2; RVM, rostral ventromedial medulla; SCFA, short chain fatty acid; ST25, tianshu; ST36, zusanli; ST37, shangjuxu. The figure was created with BioRender.com.

Research on FGIDs (neural circuitry dynamics), psychiatric conditions (neuro‐immune‐endocrine integration), and chronic pain (humoral network modulation) establishes acupuncture as a master regulator of central‐peripheral communication networks. Rather than targeting isolated pathology, it restores dynamic equilibrium across hierarchically organized physiological systems through three cardinal mechanisms: brain‐gut/sensory axis regulation, cytokine‐HPA‐glial signaling, hormone/metabolite‐mediated cross‐talk (Figure 3). Looking forward, the convergence of connectomics, epigenetic profiling, and computational neuroimaging will enable precision acupuncture protocols tailored to individual neural‐immune phenotypes. This evolution positions acupuncture as a vanguard approach in integrative neurology. Translating ancient wisdom through modern neuroscience, we stand at the threshold of an era where needle stimulation joins the arsenal of targeted neuromodulation, revolutionizing our management of psychosomatic disease complexity.

## 3 Translational Outlook: Designing Next‐Generation Clinical Trials

Building on the mechanistic framework, we propose actionable clinical trial designs targeting unresolved challenges. Future clinical trials should adopt three core principles: (1) Mechanism‐informed design with target engagement verification (e.g., fMRI for neural pathway trials); (2) Precision stratification using biomarkers (e.g., serum BDNF for depression cohorts); (3) Comparator rigor employing active vs. sham acupuncture with optimized blinding. Methodologically, these studies must overcome placebo control challenges inherent to physical interventions and strictly adhere to STRICTA guidelines for protocol standardization.

## 4 Challenges and Future Perspectives

While acupuncture exhibits a favorable safety profile with rare severe adverse events [109] (≤ 0.1%), practitioners must address common minor complications—predominantly needle site bleeding (3.1%) and transient pain (1.1%) [110]—through enhanced training and standardized protocols. Critical challenges persist in clinical validation: (1) *Methodological Rigor*. Sham controls using non‐insertive needles may inadvertently activate cutaneous C‐mechanoreceptors, confounding outcomes. (2) *Heterogeneity Issues*. Negative findings in conditions highlight the need for phenotype‐specific protocols [111]. (3) *Mechanistic Gaps*. Despite clinical efficacy in pain and FGIDs, limited translatability between animal models and human neuroimmune responses obscures target identification. Our solutions are as follows: (1) *Precision Trial Design*. Multi‐arm studies comparing acupoint specificity, stimulation parameters, and treatment duration. (2) *Omics‐Driven Insights*. Leverage neuroimaging (fMRI/PET) and single‐cell sequencing to map acupoint‐brain‐organ axes. (3) *AI‐Enhanced Practice*. Develop decision‐support systems aligning needling strategies with individual genetic/epigenetic profiles. By harmonizing TCM's holistic principles with mechanistic rigor, acupuncture can evolve into a reproducible, data‐driven modality for personalized neuromodulation.

## Author Contributions

L.Z. and Y.L.: conceptualization, visualization, writing – original draft. T.W. and D.W.: visualization (Figure Creation). Z.H.: formal analysis (table design and development). L.L. and F.Z.: conceptualization, writing – review and editing, supervision.

## Ethics Statement

The authors have nothing to report.

## Consent

Consent to publish has been obtained from all authors.

## Conflicts of Interest

The authors declare no conflicts of interest.

## Data Availability

The authors have nothing to report.
